# Policy strategies for inclusion of pregnant and lactating women in vaccine research

**DOI:** 10.1093/haschl/qxaf035

**Published:** 2025-02-19

**Authors:** Danielle E Serota, Chelsea M Crooks, Emma E McGinty, Sallie R Permar, Laura Riley

**Affiliations:** Washington University School of Medicine, St. Louis, MO 63130, United States; Department of Pediatrics, Weill Cornell Medicine, New York, NY 10065, United States; Division of Health Policy and Economics, Department of Population Health Sciences, Weill Cornell Medicine, New York, NY 10022, United States; Department of Pediatrics, Weill Cornell Medicine, New York, NY 10065, United States; Department of Obstetrics and Gynecology, Weill Cornell Medicine, New York, NY 10065, United States

**Keywords:** pregnancy, lactation, vaccine, clinical research

## Abstract

Pregnant and lactating women (PLW) have historically been excluded from vaccine research, creating inequities in vaccine access and uptake for PLW. US federal research policies create the framework for inclusion of diverse populations, including PLW, in research. We conducted a policy analysis and interviews (*n* = 29) with experts in vaccine research for PLW to characterize the existing policy landscape and to identify policy strategies to promote inclusion of PLW in vaccine trials. We integrated policy analysis and interview results to inform 5 policy recommendations. Current federal policy does not require or incentivize the inclusion of PLW in vaccine trials. The Food and Drug Administration (FDA) guidance on inclusion of PLW in vaccine and other clinical research is nonbinding and lacks clarity. Extant policies do not adequately allay liability concerns among vaccine developers. To address these concerns, we recommend that US Congress pass legislation authorizing the FDA to require inclusion of PLW in vaccine research; incentivize sponsors to complete timely vaccine studies in PLW; create a national no-fault compensation program for clinical research participants, including PLW; remove pregnancy from the “vulnerable population” designation in FDA human subjects regulations; and clarify existing federal regulations pertaining to clinical research with PLW.

## Introduction

Pregnant and lactating women (PLW) have historically been excluded from vaccine research, posing significant health risks for these populations and the next generation.^1-3^ In the United States, over 3.5 million people give birth annually and nearly 3 million begin breastfeeding each year.^4,5^ Pregnant and lactating women often face heightened risk from vaccine-preventable diseases like influenza, Ebola, and COVID-19, leading to adverse pregnancy outcomes.^6-8^ Yet, PLW are typically excluded from early-phase vaccine development. During the COVID-19 pandemic, exclusion of PLW from early vaccine research contributed to low vaccine uptake (45% among pregnant women)^9^ among pregnant women^10^

US federal policies create the framework for inclusion of diverse populations in research.^11^ While extant policies operate on the principle that populations included in vaccine research should be as diverse as the population who will use the vaccine, PLW are typically excluded.^12^ Confusion surrounding human subjects research policies that set the parameters for ethical inclusion of PLW in research; fears of liability for vaccine-related injuries incurred by PLW, or their fetus/child, while participating in vaccine research; and lack of financial incentive for pharmaceutical companies to study vaccines among PLW have been identified as barriers to including PLW in vaccine studies.^2,12,13^ However, it is unclear how the current policy framework drives these barriers or how policy changes could mitigate them.

The 2018 Federal Task Force for Research Specific to Pregnant and Lactating Women (PRGLAC)^14^ and the National Academies of Science, Engineering, and Medicine (NASEM) Committee on Developing a Framework to Address Legal, Ethical, Regulatory, and Policy Issues for Research Specific to Pregnant and Lactating Persons, which issued its final report in 2024,^12^ have made recommendations regarding the inclusion of PLW in clinical research including, but not limited to, policy changes. Therefore, we conducted a policy analysis and solicited expert opinion designed to answer these questions. Our objectives were to (1) describe the existing US policy landscape surrounding inclusion of PLW in vaccine research, (2) characterize expert perceptions of US policy changes needed to increase inclusion, and (3) make new policy recommendations based on findings from the policy analysis and expert interviews. We focused on US policy. While inclusion of PLW in vaccine trials is a global issue,^3,15^ a comprehensive set of recommendations across policy contexts worldwide is out of the scope of this study.

## Data and methods

### Policy analysis

We conducted a policy analysis with 2 objectives. First, to characterize the current US federal policy landscape surrounding inclusion of PLW in vaccine research. Second, to identify existing federal policies that could be used as models for policies to support early inclusion of PLW in vaccine research. Policy models were defined as policies designed to (1) increase inclusion of other traditionally excluded populations in clinical research (eg, children) or to (2) incentivize vaccine or pharmaceutical development for specific conditions (eg, rare diseases). For each policy model identified, we summarized available evidence on whether the policy achieved its goals.

We abstracted policies referenced in the 2018 PRGLAC Task Force report.^14^ We searched the internet and scholarly databases (PubMed, Google Scholar) to identify policies, reports, and peer-reviewed literature addressing issues identified in the PRGLAC report or other policies relevant to our objectives. We summarized findings using an integrative review approach^16^ to synthesize evidence produced through diverse methodologies. The review was conducted during October 2022–March 2023 and was updated in September 2024. The search strategy and abstraction tool for detailing each policy and its related literature are detailed in Appendix S1.

### Expert interviews

We interviewed 29 experts whose professional roles involved vaccine development, clinical research policy, and/or clinical research involving PLW. Interviews were conducted via videoconference from February through September 2023, with a median interview duration of 57 minutes (range: 29–71 minutes).

### Participant recruitment

Our research team identified 46 experts with experience serving in relevant roles at federal agencies or on boards, task forces, or commissions charged by the federal government with making recommendations in these domains in 2018 or later. Of these, 26 experts agreed to an interview (4 declined and 16 did not respond). The initial interviewees recommended an additional 3 experts who completed interviews, for 29 interviews total. The 29 experts included interviewees whose primary professional affiliations at the time of the interview were with federal agencies (*n* = 6), academic institutions (*n* = 8), health systems (*n* = 7), professional or other nongovernmental organizations (*n* = 4), and pharmaceutical companies (*n* = 4). (See Appendix S2 for a description of interviewees’ professional experience and expertise, geographic region, and sex.) Interview recruitment was conducted via email and LinkedIn.

### Interview administration

After obtaining oral consent, 2 team members conducted interviews using a semi-structured interview guide (Appendix S3), with 1 team member leading the interview and the other taking notes and asking follow-up questions. The guide was designed to elicit experts’ perceptions of and preferences for policy options to increase inclusion of PLW in vaccine trials. With consent, interviews were recorded and transcribed. Immediately after each interview, the lead interviewer created a memo summarizing major themes. The themes in the memos were used to develop the coding framework, which was applied using a content analysis approach^17^ to summarize key themes across interviews. This study was approved by the Weill Cornell Medical College Institutional Review Board.

## Results

### Current policy landscape

The Department of Health and Human Services (HHS), which oversees agencies related to public health, welfare, and social services, and 2 of its agencies, the US Food and Drug Administration (FDA) and the National Institutes of Health (NIH) have primary responsibility for creating and implementing the policies that shape inclusion of PLW in vaccine research. In addition, the Centers for Disease Control and Prevention (CDC), specifically the CDC's Advisory Committee on Immunization Practices (ACIP),^18^ recommends vaccines for use by PLW.

#### Federal policy for the protection of human subjects

The HHS issues federal regulations for the protection of human subjects. These regulations, 45 CFR (Code of Federal Regulations) 46—the “Common Rule”—have 4 subparts.^19^ Subpart A describes protections for all human subjects and subparts B, C, and D delineate additional protections for pregnant women, human fetuses, and neonates (B), prisoners (C), and children (D). The original regulations, promulgated in 1991, deemed pregnant women a “vulnerable population” open to undue coercion or influence in their decision to consent to participate in a clinical trial. This designation was removed in 2018, and current HHS regulations allow pregnant women to be included in clinical research as long as 10 conditions are met, including that risk to the fetus is caused solely by interventions/procedures that could have direct benefit to the pregnant person or the fetus or, if no such benefit is expected, that the risk to the fetus is “not greater than minimal.”^19^ Subpart B 46.204(e) requires the consent of both the pregnant person and the father if the research has the prospect for direct benefit solely to the fetus. Lactating people are not mentioned in the Common Rule.

While most federal agencies are signatories to the Common Rule, the FDA has additional human subjects research regulations (21 CFR 50).^20^ Although the 2016 21st Century Cures Act^21^ required the FDA to harmonize most of its policies with the Common Rule, as of October 2024 the FDA policy still denotes pregnant women as a “vulnerable population.” In 2022, the FDA released a Proposed Rule that would remove pregnant women as a “vulnerable population”; however, it has not been finalized.^22^

#### Federal vaccine licensing policy

The FDA licenses vaccines for use in a specified population after scientific review concluding that the vaccine's known and possible benefits outweigh the known and possible risks.^23^ The FDA is also responsible for surveillance of the safety and effectiveness of vaccines after they are distributed to the intended population.^24^ The FDA does not have the authority to require the pharmaceutical companies sponsoring vaccine-development research to include PLW. However, in 2018, the FDA released draft guidance titled “Pregnant Women: Scientific and Ethical Considerations for Inclusion in Clinical Trials,”^25^ which provided recommendations for the inclusion of pregnant women in clinical trials for drugs and biologics. In 2019, the FDA released an additional draft guidance titled “Clinical Lactation Studies: Considerations for Study Design,”^26^ which provided recommendations for the collection of robust data on drug exposure through breast milk to inform safe medication use for lactating people and their infants. However, as of February 2025, neither draft guidance was finalized.

#### Federally funded clinical research policy

The NIH sets inclusion policies detailing which subpopulations are expected to be included in NIH-funded research studies. The NIH Revitalization Act of 1993^27^ required the NIH to establish guidelines for including women and minorities in research. No specific guidelines for the inclusion of PLW were included. While this Act enhanced inclusion of these groups overall, women are still underrepresented in many studies.^28,29^ The 2016 21st Century Cures Act^21^ required the NIH to update these guidelines to reflect individuals of all ages. Again, no specific guidance on PLW was included.

## Federal task forces and committees

The 2016 21st Century Cures Act^21^ established the PRGLAC, a federal advisory committee tasked with addressing the lack of research on the safety and efficacy of therapeutic products for PLW.^30^ In 2018, the PRGLAC Task Force made 15 recommendations, 2 of which focused on changes to the Common Rule regulations.^14^ These included (1) removal of pregnant women as an example of a vulnerable population in the Common Rule and (2) modification of the Common Rule subpart B to allow for maternal consent alone and to allow for research posing “minor increase over minimal risk” to the fetus (vs “minimal risk”).^14^ In 2020, PRGLAC submitted an Implementation Plan to Congress,^31^ and in 2023, the National Institute of Child Health and Human Development (NICHD) established the PRGLAC Implementation Working Group in Council to monitor and facilitate implementation.^32^ As of July 2024, 2 of the original 15 recommendations have been fully implemented, including the Common Rule vulnerable population recommendation above and a recommendation to extend the duration of government-funded grants supporting research with PLW.^33^

In January 2023, the NASEM, which operates under a congressional charter but remains independent with the goal of providing nonpartisan, evidence-based guidance, convened the Committee on Developing a Framework to Address Legal, Ethical, Regulatory, and Policy Issues for Research Specific to Pregnant and Lactating Persons. The Committee's April 2024 consensus report made 9 recommendations for PLW,^12^ 2 of which focused on enacting federal laws modeled on the Best Pharmaceuticals for Children Act (BPCA)^34^ and the Pediatric Research Equity Act (PREA), discussed below.^35^ Notably, the NASEM committee found little evidence to support legal liability concerns.

### Model policies

We identified 4 policy models meeting the definition above. Available evidence suggests that all 4 of these federal policies have at least partially achieved their goals (see Table 1 for details).

**Table 1.** Policies that could be used as models for increasing inclusion of pregnant and lactating women (PLW) in vaccine research.

Policy	Policy overview	Application(s)	Evidence summary^a^
Existing policy models			
Pediatric Research Equity Act (PREA) of 2002	Grants FDA authority to mandate pediatric studies of vaccines and other products. Sponsors of studies must provide FDA with data on the product's safety and effectiveness in children or provide justification for extrapolating data from adult studies to relevant pediatric subpopulations for the indications being reviewed in adults.	Pediatrics	FDA data from March 2024 state that 876 products gained pediatric-specific labeling based on pediatric studies conducted as a result of PREA alone (*n* = 706) or a combination of PREA and BPCA (*n* = 170) between 2002 and February 2024.^36^ From 2007–2014, the FDA approved 114 total new drugs (*n* = 84) and new indications for already approved drugs (*n* = 30) subject to PREA^37^; 41% of these drugs had labels containing pediatric efficacy, safety, or dosing information.^37^ FDA required 222 pediatric post-marketing clinical studies for these 114 approved drugs: 27% of these 22 studies were completed within 5 years of FDA approval.^37^
Best Pharmaceuticals for Children Act (BPCA) of 2003	Provides an additional 6 months of market exclusivity to sponsors who perform pediatric studies of vaccines and other products, including those in development and on-patent products approved by the FDA but lacking data to inform dosing, safety, and effectiveness in children. Authorizes NIH to prioritize study of off-patent drugs that require further study in children.	Pediatrics	FDA data from March 2024 states that 391 products gained pediatric-specific labeling based on pediatric studies conducted as a result of BPCA alone (*n* = 221) or a combination of BPCA and PREA (*n* = 170) between 2003 and February 2024.^36^ BPCA has led to NIH prioritizing over 200 drugs/therapeutics (with a focus on off-patent products) and 50 conditions for further study.^38^ NIH has funded 45 pediatric clinical trials of these priority products, resulting in 24 labeling changes.^39^
Vaccine Injury Compensation Program (VICP), created by the National Child Vaccine Injury Act of 1986	Established a no-fault liability compensation program for resolving vaccine injury petitions outside of the traditional tort system (ie, a lawsuit alternative). Funded by an excise tax on covered vaccines. VICP applies to post-market vaccines; it does not provide liability protection for injuries sustained by participants in vaccine trials.	All populations that the CDC recommends routinely receive a given vaccine, including children and pregnant women	From 1988 through August 2024, the VICP received more than 27 102 claims, of which 24 050 were adjudicated. Of those, 11 233 resulted in compensation and 12 817 were dismissed.^40^ Estimated total compensation paid over this period is $5 billion.^40^ The VICP is widely recognized as a successful model for managing the post-market risks of vaccines in the United States.^41^
Orphan Drug Act (ODA) of 1983	Provides financial incentives for the research and development of therapeutics for rare diseases. Incentives include federal tax credits of up to 25% (previously 50%^42^) of the total research and development (R&D) qualified research expenses each year, waiver of FDA approval fees; 7-year market exclusivity; and priority review vouchers.	Rare diseases affecting fewer than 200 000 people, or diseases affecting more than 200 000 people for which drug-development costs will not be recouped by sales	As of October 1, 2024, there have been 7322 orphan drug designations and there are 1252 orphan drug indications approved.^43^ Orphan drug designations nearly tripled between the 2000s and 2010s.^44,45^

^a^Summary of the most recent available evidence identified in the integrative review.

Abbreviations: CDC, Centers for Disease Control and Prevention; FDA, Food and Drug Administration; NIH, National Institutes of Health.

#### PREA

The PREA,^35^ enacted in 2003, gives the FDA the authority to require inclusion of children in research on vaccines, drugs, and medical devices, including studies assessing the safety and efficacy of new vaccines or studies of new indications, dosages, and routes of administration of previously approved products. For new products, PREA requires sponsors to conduct pediatric studies before the product can be approved, thus providing patients and clinicians with pediatric data as soon as the product becomes available.^37^ Still, pharmaceutical companies are allowed to request deferrals until after approval, and delays in pediatric data are common (see Table 1).^37^ The FDA can waive the pediatric study requirement if the disease to be treated occurs primarily in adults or if existing evidence suggests that the drug is ineffective or unsafe in children.^37,40^

#### BPCA

The BPCA, enacted in 2002,^34^ incentivizes research on vaccines and other FDA-licensed products by providing an additional 6 months of exclusivity to industry sponsors when they include children in early-phase product-development studies. The incentive also applies to studies to obtain safety and effectiveness data for children of on-patent products.^46^ This exclusivity incentive applies to studies required under PREA and is predicated on reporting data within a specific timeframe.

The BPCA also authorizes the NIH to prioritize research on off-patent products lacking data for children. The NIH implements this requirement by identifying pharmaceuticals for study, sponsoring clinical trials, and submitting study data to the FDA, so that FDA can use the data to inform labeling modifications (eg, labeling regarding the appropriate dose in children).^47^

#### The Vaccine Injury Compensation Program

The National Childhood Vaccine Injury Act of 1986 established the Vaccine Injury Compensation Program (VICP),^48^ which shields vaccine manufacturers and administering providers from liability by providing a no-fault alternative to the traditional tort system for addressing vaccine injuries. The VICP is funded by an excise tax on covered vaccines. In 2016, the 21st Century Cures Act^21^ expanded VICP's coverage to include vaccines recommended by the CDC for routine administration to pregnant women. The Cures Act clarified that vaccine-injury claims may be filed for injuries alleged to have been sustained by liveborn children who were in utero when the vaccine was administered. Importantly, VICP liability protections are only for post-market vaccines; VICP does not provide liability protection for injuries sustained by participants in vaccine trials.

In addition, the Public Readiness and Emergency Preparedness (PREP) Act of 2005^49^ allows the Secretary of HHS to issue a declaration providing immunity from tort liability for vaccines developed in response to a public health emergency and authorizes compensation by the Countermeasures Injury Compensation Program (CICP) for individuals injured by vaccines in this scenario.^50^ The CICP can apply to vaccines in development. Despite invocation of the PREP Act by HHS in response to COVID-19, PLW were still excluded from early-stage vaccine trials.

#### Orphan Drug Act

The Orphan Drug Act of 1983^51^ incentivizes pharmaceutical companies to develop vaccines or drugs targeting (1) rare diseases affecting fewer than 200 000 people or (2) diseases that affect more than 200 000 people for which there is no reasonable expectation that the pharmaceutical company will recoup drug-development costs. Pharmaceutical manufacturers developing a product with the orphan drug designation get tax credits of up to 25% (previously, 50%^42^) of the total research and development expenses; a waiver of FDA approval fees; a 7-year market exclusivity period, defined as the period in which the FDA cannot approve any other treatments for the same disease; and priority review vouchers allowing the company to receive priority review for a different product in the future.^51,52^

### Expert interviews

The following 6 themes emerged from the expert interviews, described below and summarized in Table 2 alongside illustrative quotes.

**Table 2.** Expert perceptions of policy strategies to improve inclusion of pregnant and lactating women (PLW) in vaccine research.

Policy strategies
**A policy framework should require early inclusion of PLW in vaccine research.** Experts viewed requiring early inclusion of PLW in vaccine development research as a needed policy strategy, and frequently cited the Pediatric Research Equity Act (PREA), which gave the FDA authority to require inclusion of children in vaccine research, as a model.
*Illustrative quotes:*
“Well, right now, you know right now it's up to [pharmaceutical] companies right? It's their choice. It's their decision. There are no legal requirements that this [inclusion of PLW] happens. So it's the company choice, and regulators can suggest. But usually, typically, at least in the US, unless there's a law. And we can talk about PREA, I'm sure you're familiar with that.”
“There probably would need to be a law because absent, a law, you know the NIH and the FDA could say, ‘We think this is a good idea, but you know, how would they require it? I mean their funding of vaccine trials is minuscule relative to private companies.’”
**A policy framework should incentive additional vaccine research among PLW.** Experts noted that a PREA-like policy requiring inclusion of PLW in vaccine research should be accompanied by a policy incentivizing the conduct of such research, along the lines of the Best Pharmaceuticals for Children Act (BPCA) that provides an additional 6 months of exclusivity to industry sponsors when they conduct pediatric studies in a timely manner, including studies requiring inclusion of children under PREA.
*Illustrative quotes:*
“But you know there is the Best Pharmaceuticals for Children Act/PREA kind of combo on the pediatric side it's been really effective…. I think it's hard not to look at that model and think, wow! We could really benefit from a similar thing over here.”
“The pediatric laws—unless there's a law—regulatory agencies have a hard time saying, ‘Oh, we think it might be a good idea for you to do this.’ They're more on the receiving end. They receive information, but they can't necessarily, unless there's a particular scientific reason, they don't have the ability to say, oh, you know, we think it would really be good to include pregnant women. Yeah, so we have, BPCA, primarily, PREA, and how that could be used as a model for a potential legislation for pregnant women.”
**Policy to address liability concerns is needed.** Experts described liability concerns as a major barrier to inclusion of PLW in vaccine research. To address liability, they discussed options including a national no-fault compensation program for participants in vaccine trials, citing the Vaccine Injury Compensation Program (VICP), which offers protections for post-market vaccines. Experts also discussed clinical trials insurance as an option to mitigate liability concerns.
*Illustrative quotes:*
“But one thing I think that is always helpful in the legislative process is precedent and existing programs that have been successful. And so I think that vaccine compensation program is a really good model. I think we'd want to tread a little bit carefully in terms of messaging it in terms of like suggesting that someone would be injured or harmed right? So there'll be need to be some messaging around that. But I think in terms of a program that has, like been viewed as generally successful, that has bipartisan support that has served an important purpose. I think that's always a good model.”
“I think some of the bigger challenges are thinking about trial insurance and how to protect institutions and pharmaceutical companies from liability, or to convince them that doing the trial is not going to result in untenable liability.”
**Existing policies need clarification and improved implementation.**
*Illustrative quotes:*
“Actually, in many cases, subpart B is permissive around inclusion, so long as there is the prospect of direct benefit to the pregnant women, the fetus or both, then, that you are allowed to have some level of risk as though as well as the risk–benefit ratio is acceptable. Different people will view acceptability differently, depending on who they are…. There is a lot of ambiguity in prospective direct benefit research. That is a matter for debate, and you know, Institutional Review Boards and others tend to err on the side of caution, better safe than sorry.”
“Also in studies that do not hold the prospect of direct benefit, the risk is capped at minimal risk and minimal risk is a very vexed concept, and for a variety of reasons. But when you think about minimal risk in the context of pregnancy, there is a tendency to view any risk even an imagined risk as more than minimal. So if you don’t know if there's any risk or you think there's probably not, but there could be, the presumption is that it is more than minimal, because risk in pregnancy is taboo.”
**Policies should distinguish between pregnant and lactating populations.**
*Illustrative quotes:*
“There's sort of this knee-jerk response when people are writing clinical protocols to say exclusion criterion, you know, pregnant and lactating women, and it's without even sort of consideration of what is the biology? What are your concerns…they actually don't think about lactating people much, even though, if you think about it, somebody is likely to be, may well be lactating for much more of their lives than they are pregnant, you know. I mean, it ends up being a bigger issue.”
“It doesn't actually serve lactating people well to be grouped with pregnant women, because also those considerations are completely different. And they are actually the ones who always get forgotten and left behind. And we certainly know that with Covid, where there really were not good reasons to not include lactating people from the outset, and yet they were not included. So considerations should be really very different.”
**A central unit for PLW vaccine and other clinical research is needed to implement policies.**
*Illustrative quotes:*
“The other thing that comes into play is that pregnant and lactating women and people do not actually have a place that they are designated for research focus. NICHD is about child and human development. It's not about pregnancy.”
“Well, the question becomes, so there is, it's called an Office of Research for Women's Health, but that's an office, not a focused institute or center. That's fine except that it's across the board for women's health, not specifically women's reproductive health.”

Abbreviation: NICHD, National Institute of Child Health and Human Development.

#### A policy framework should require early inclusion of PLW in vaccine research.

Experts consistently perceived passing federal legislation to require early inclusion of PLW in vaccine and other clinical trials as a needed policy strategy.

#### A policy framework should incentivize additional vaccine research among PLW.

Experts noted that PREA-like federal policy requiring inclusion of PLW in vaccine-development research should be accompanied by BPCA-like federal policy incentivizing pharmaceutical companies and the NIH to conduct PLW-focused studies of vaccines—a “carrot” to accompany the “stick” of PREA. Several experts suggested considering incentives beyond the BPCA's 6-month extended exclusivity period. Experts noted that extended exclusivity is more valuable to smaller pharmaceutical companies than the larger companies that typically manufacture vaccines. Interviewees suggested that tax credits and priority review vouchers, both used in the Orphan Drug Act, could be considered.

#### Policy to address industry liability concerns is needed.

Interviewees cited industry liability concerns as a major barrier to the inclusion of PLW in clinical research and vaccine trials specifically and suggested 2 types of policies that could address liability concerns. First, develop a VICP-like policy implementing a no-fault alternative to traditional lawsuits in the tort system that compensates research participants for injuries stemming from use of the vaccine. Second, establish policies requiring and/or funding clinical trial insurance to protect against injuries resulting from trial participation. Interviewees noted that, currently, the United States has no policies requiring clinical trials insurance or programs to cover or subsidize higher insurance costs for trials studying special populations.

#### Existing policies need clarification and improved implementation.

Experts emphasized that misinterpretation of current federal policies impedes early inclusion of PLW in vaccine research. Experts noted that subpart B of the Common Rule permits the inclusion of pregnant women if there is a potential direct benefit to them or the fetus or has no more than minimal risk to the fetus. Experts described this rule as commonly misinterpreted by industry sponsors as well as institutional review boards (IRBs). Interviewees viewed these entities as erring on the side of caution due to ambiguity in defining “direct benefits” and “not greater than minimal” risk. Interviewees discussed the need to strengthen IRBs by including obstetric education and experts on review boards. Although some experts suggested that IRBs should require justification from sponsors for the exclusion of PLW, others believed that this would exceed the IRBs’ responsibilities.

#### Policies should distinguish between pregnant and lactating populations.

Interviewees consistently noted that policies need to distinguish between pregnant and lactating populations, which require distinct trial designs and risk–benefit considerations. Interviewees asserted that policies need to clearly delineate rules surrounding inclusion of each of these 2 populations in vaccine trials rather than combining them.

#### A central unit for PLW vaccine and other clinical research is needed to implement policies.

Experts identified the lack of a unit responsible for PLW research conduct and oversight as a barrier to policy implementation and accountability.

## Policy recommendations

The present US vaccine policy landscape has led to consistent under-inclusion of PLW in vaccine research, with significant public health implications. The policy recommendations summarized in Table 3 are discussed below. Within each policy, rules and guidance for PLW should be delineated, recognizing the unique considerations of each group. In addition, a single government unit dedicated to clinical research including vaccines for PLW is needed to ensure implementation and accountability for these policies. As we have seen with PREA implementation and delays in pediatric data reporting,^46^ we will not reap the full benefit of these policies that aim to reduce maternal and fetal morbidity and mortality unless there is robust implementation and enforcement.

**Table 3.** Policy recommendations to increase inclusion of pregnant and lactating women (PLW) in vaccine research.

Policy recommendations
1. The US Congress should pass legislation, modeled on PREA, to allow FDA to require inclusion of PLW in research on vaccines and other treatments regulated by the FDA, including drugs and medical devices.
2. The US Congress should pass legislation, modeled on BPCA, to incentivize early inclusion of PLW in vaccine development research and in additional research on vaccines approved for the general population but lacking robust data for PLW. Data from this research should be used to provide additional information about dosing, safety, and efficacy for PLW in product labeling.
3. The US Congress should pass legislation, modeled on the National Childhood Vaccine Injury Act that created the VICP program, to create a no-fault compensation program for clinical research participants, including PLW. The program should provide a no-fault alternative to lawsuits for injuries incurred from vaccines or other treatments being tested in clinical research.
4. The US Congress should pass legislation requiring the FDA to harmonize its human subjects research policies with the HHS Common Rule, removing the “vulnerable population” designation for pregnant women. This designation is for populations open to undue coercion or influence in their decision to participate in clinical research and as a result requires special protections, which in often limits inclusion in clinical research.
5. The HHS and FDA should clarify current policies relevant to inclusion of PLW in vaccine trials. HHS should clarify Common Rule definitions of “direct benefit” to pregnant persons or the fetus and “not greater than minimal” risk to the fetus as these terms determine the conditions under which pregnant persons can be included in vaccine trials. The FDA should clarify its guidance on including PLW in vaccine trials to specifically state an expectation that PLW be included as early as possible.
Cross-cutting recommendation: Policies should encompass both pregnant and lactating populations but separately delineate rules for them.

Abbreviations: BPCA, Best Pharmaceuticals for Children Act; HHS, Department of Health and Human Services; FDA, Food and Drug Administration; PREA, Pediatric Research Equity Act; VICP, Vaccine Injury Compensation Program.

We compared our policy recommendations to those issued by the PRGLAC Task Force and the NASEM Committee on Developing a Framework to Address Legal, Ethical, Regulatory, and Policy Issues for Research Specific to PLW.^12^ While we focus on these sources as they are the most recent US-specific initiatives, it is important to note that their recommendations were informed by broader global initiatives, such as the COVAX Maternal Immunization Working Group and the Pregnancy Research Ethics for Vaccines, Epidemics, and New Technologies (PREVENT) guidance for inclusion of PLW in vaccine research.^3,15,53,54^

First, the US Congress should pass legislation, modeled on PREA, to allow FDA to require inclusion of PLW in research on vaccines. Adaptations from the PREA model will be needed to reflect the unique realities of research with PLW, such as the need for Developmental and Reproductive Toxicology (DART) studies to assess potential fetal effects before human trials.^55^ As DART studies are time-consuming and costly, vaccine developers have an incentive to wait until after they have significant positive data in the general population—suggesting the product is likely to go to market—before conducting DART studies. A PREA-type policy will need to address this issue, potentially by setting deadlines for completion of DART studies in addition to in-human trials. Designers of a PLW-focused policy should consider how to overcome study delays and inadequate results reporting observed in PREA implementation^46^ via policy design (eg, tying incentives to timely results reporting) and/or heightened oversight and enforcement authority by the FDA. This recommendation to pass legislation to require inclusion of PLW in research on vaccines was also made by the NASEM Committee.

Second, the US Congress should pass legislation, modeled on BPCA, to incentivize early inclusion of PLW in vaccine-development research and support research on vaccines approved for the general population but lacking robust data for PLW. Federal policy allowing the FDA to require inclusion of PLW in vaccine research should be accompanied by BPCA-like policy incentivizing industry sponsors to include PLW in early-stage research and report data in a timely manner. In addition, policy is needed to generate PLW-specific data on dosing, safety, and efficacy of vaccines approved for use in the general population. Incentives should be aligned to benefit sponsors and may exceed the extended exclusivity included in BPCA to encompass additional incentives such as tax credits and priority review vouchers. This recommendation was also made by the NASEM Committee.^12^ The PRGLAC Task Force's implementation plan recommended reviewing federal program experiences, including BPCA, to determine successful approaches and also recommended development of separate programs to study therapeutic products used off-patent in PLW, with BPCA as a model.^31^

Third, the US Congress should pass legislation, modeled on the National Childhood Vaccine Injury Act that created the VICP program, to create a national no-fault compensation program for clinical research participants, including PLW. A VICP-like program should be created for clinical research participants, including but not limited to PLW, to provide a no-fault alternative to lawsuits for injuries incurred from the vaccine or other treatment under study. The NASEM Committee stopped short of recommending such a program, deeming it outside their scope, which was limited to PLW.^12^ However, their final report noted that they favored such a program for all clinical research participants. The NASEM Committee recommended that the NIH and other federal agencies funding clinical research cover the cost of clinical trial insurance for grant-funded trials involving PLW.^12^ We concur with this recommendation but view a national no-fault compensation program as a more impactful policy solution for early inclusion of PLW in vaccine-development research, given that most such research is funded by pharmaceutical companies.

Fourth, the US Congress should pass legislation requiring the FDA to harmonize its human subjects policies with the HHS Common Rule removal of pregnant women as a “vulnerable population.” Persistence of the “vulnerable population” designation in the FDA regulations furthers the misconception, among sponsors and IRBs, that pregnant women should generally not be included in early-stage vaccine trials. This recommendation was also made by the PRGLAC Task Force.^14,31^ The Advancing Safe Medications for Moms and Babies Act,^56^ currently being considered by US Congress, would require harmonization.

Fifth, the HHS and FDA should clarify current policies relevant to inclusion of PLW in vaccine trials. The HHS should clarify Common Rule definitions of “direct benefit” to pregnant women or the fetus and not greater than “minimal risk” to the fetus as these terms determine the conditions under which pregnant women can be included in vaccine trials. Both the NASEM Committee and PRGLAC recommended that HHS clarify the “minimal risk” terminology^12,31^; the NASEM Committee also recommended clarification of what “additional safeguards” need to be put in place for responsible and ethnical research with PLW.^12^

## Limitations

Interviews were conducted with a convenience sample of experts. To mitigate potential researcher biases in the interpretation of results, we used a semi-structured interview guide and coding process. Policy recommendations are based on our team's interpretation of the policy analysis and interview results and should not be construed as consensus recommendations among the experts interviewed; not all interviewees suggested all policies and no formal consensus process was conducted. Future research should include the perspectives of PLW on this issue.

## Conclusion

As the number of infectious disease threats grows across the globe, current vaccine approval practices will continue to leave PLW and their offspring at disparate risk of lasting health consequences from vaccine-preventable diseases without policy changes that require and incentivize testing of vaccines in these important populations. Existing initiatives to improve inclusion have been hindered by lack of a supportive policy framework.^3,12,14,15,54^ By requiring and incentivizing the early inclusion of PLW in vaccine research, addressing liability concerns, and clarifying regulatory guidelines, these policy recommendations can close the gap in vaccine development for PLW. It will be critical to address public concerns and foster trust to ensure broader acceptance of policy solutions. Most vaccine trials are sponsored by companies in the United States and other high-income countries, leading to vaccines that may not be optimized for local needs in the global South.^57^ Global initiatives to improve inclusion of PLW in vaccine research in these contexts are crucial.^3,12,15,53,58^

## Supplementary Material

qxaf035_Supplementary_Data

## Data Availability

Policy analysis and qualitative interview theme data are presented in the manuscript. Qualitative interview data cannot be shared due to confidentiality protections.
