# Supplementary material for: Policy strategies for inclusion of pregnant and lactating women in vaccine research
Source: Health Aff Sch. 2025 Feb 19;3(3):qxaf035. doi: 10.1093/haschl/qxaf035 (PMC11891035; doi:10.1093/haschl/qxaf035)
Supplement: qxaf035_Supplementary_Data [file qxaf035_supplementary_data.zip › Supplement.docx]

**Appendix 1.**

**Search Strategy**

Web (using the Google search engine), *Google Scholar*, and *PubMed* were searched from inception to September 2024 for relevant studies. The search used the following terms (with synonyms and closely related words):

- “Pregnant” AND “vaccine” AND [“trial”/“research”/”study”] AND “policy”
- “Lactate” AND “vaccine” AND [“trial”/“research”/”study”] AND “policy”

Reference lists of materials identified through the initial search were used to identify additional content. The searches were limited by English language.

**Abstraction Rubrics**

Landscape analysis

Policy documents (e.g., law test, Congressional Research Service summaries) reports, articles, or commentaries describing the current policy landscape shaping inclusion of pregnant and lactating people in vaccine research:

| Policy Domain | Citation | Policy overview | Key provisions related to including PLP in vaccine research |
| --- | --- | --- | --- |
| Policy #1 |  |  |  |
| Document/report 1 |  |  |  |
| Document/report 2 |  |  |  |
| Etc. |  |  |  |
| Policy #2…X |  |  |  |

Model policy analysis

Policy documents (e.g., law test, Congressional Research Service summaries) reports, articles, or commentaries describing policies that have increased inclusion of specific groups in vaccine or pharmaceutical research, or incentivized vaccine or pharmaceutical research for specific conditions

| Policy Domain | Citation | Policy overview | Key provisions relevant to increasing inclusion of specific groups/incentivizing research on products for specific conditions |
| --- | --- | --- | --- |
| Policy #1 |  |  |  |
| Document/report 1 |  |  |  |
| Document/report 2 |  |  |  |
| Etc. |  |  |  |
| Policy #2…X |  |  |  |

Empirical reports or articles describing the degree to which model policies achieved their goals

| Policy name | Study population | Study outcomes | Main research question | Summary of the study’s key findings | Citation |
| --- | --- | --- | --- | --- | --- |
| Policy #1 |  |  |  |  |  |
| Report/article 1 |  |  |  |  |  |
| Report/article 2 |  |  |  |  |  |
| Etc. |  |  |  |  |  |
| Policy #2….X |  |  |  |  |  |

**Appendix 2**

**Expert interviewee’s areas of expertise**

Note that some of the 29 experts interviewed had multiple areas of expertise.

| Expertise | Number of Expert Interviewees |
| --- | --- |
| Clinical research bioethics | 3 |
| Federal vaccine research policy | 6 |
| Health equity | 2 |
| Maternal and fetal medicine | 3 |
| Women’s and/or obstetrics health policy | 4 |
| Vaccine research & development | 8 |
| Virology, infectious diseases | 6 |

**Expert interviewee’s sex**

| Sex | # Expert Interviewees |
| --- | --- |
| Female | 20 |
| Male | 9 |

**Expert interviewee’s geographical location based on US Census regions**

| Region | Division | # Interviewees |
| --- | --- | --- |
| Northeast | New England | 1 |
|  | Middle Atlantic | 8 |
| South | South Atlantic | 13 |
|  | East South Central | 2 |
|  | West South Central | 1 |
| Midwest | East North Central | 2 |
|  | West North Central | 0 |
| West | Mountain | 0 |
|  | Pacific | 2 |

**Appendix 3**

**Semi-Structured Interview Guide**

Grand Tour Questions (*These questions are designed to establish rapport with the interviewee and get their initial thoughts about the topic of interest with minimal direction from the interviewer)*

1. **Tell me about your professional role.**

*Prompts: What types of activities are you involved in? What is your main contribution(s)? How long have you been involved? Has your role changed over time?*

1. **Tell me about how your work relates to vaccine trials.**

*Prompts: How does your work relate to policy surrounding vaccine trials? How does your work relate to inclusion of vulnerable populations in vaccine trials?*

Researcher-Driven Questions (*These questions are designed to elicit interviewee responses to questions directly related to the research question(s)).*

1. **What are the barriers to including pregnant people and children in early stage vaccine trials?**

*Prompts: Do these barriers differ for pregnant people and children and if so, how? What about for pregnant people vs. lactating people? Are there existing policy barriers – in other words, current policies that get in the way of including pregnant people and/or children in early stage vaccine trials? Are there additional barriers for the inclusion of women and children of color?*

1. **What policies could be put in place to support inclusion of pregnant people and children in early stage vaccine trials?**

*Prompts: For each policy mentioned, ask:*

- *Tell me more about how exactly that policy would work.*
- *Has this policy been used in other contexts and if so, how?*
- *Are there downsides to this policy and if so, what are they?*
- *How could we design a policy that would protect women and children of color and allow them to feel safe enrolling in vaccine trials?*

1. **Of all the policies we have discussed, which do you think would be most effective at incentivizing inclusion of pregnant people and children in early stage vaccine trials, and explain why?** *Prompts: In ideal world, should multiple policies be bundled together – if so, which policies, and why? Would you prioritize policies differently for pregnant people versus children?*

1. **Are there any other thoughts you would like to share related to policies to incentivize inclusion of pregnant people and children in early stage vaccine trials?**
